# Tetra­kis(μ_2_-2-phen­oxy­propionato)-κ^3^ *O*,*O*′:*O*′;κ^3^ *O*:*O*,*O*′;κ^4^ *O*:*O*′-bis­[(1,10-phenanthroline-κ^2^ *N*,*N*′)(2-phen­oxy­propionato-κ^2^ *O*,*O*′)lanthanum(III)]

**DOI:** 10.1107/S1600536811036117

**Published:** 2011-09-14

**Authors:** Jin-Bei Shen, Jia-Lu Liu, Guo-Liang Zhao

**Affiliations:** a College of Chemistry and Life Sciences, Zhejiang Normal University, Jinhua 321004, Zhejiang, People’s Republic of China; b Zhejiang Normal University Xingzhi College, Jinhua, Zhejiang 321004, People’s Republic of China

## Abstract

In the centrosymmetric binuclear title complex, [La_2_(C_9_H_9_O_3_)_6_(C_12_H_8_N_2_)_2_], the two La(III) ions are linked by four 2-phen­oxy­propionate (*L*) groups in bi- and tridentate bridging modes. Each La^III^ ion is nine-coordinated by one 1,10-phenanthroline mol­ecule, one bidentate chelating carboxyl­ate group and four bridging carboxyl­ate groups in a distorted LaN_2_O_7_ monocapped square-anti­prismatic geometry.

## Related literature

For background to phen­oxy­alkanoic acids, see: Markus & Buser (1997 ▶). For a related La complex, see: Li *et al.* (2010 ▶). For isotypic structures, Shen *et al.* (2011*a* ▶) for Tb; Shen *et al.* (2011*b* ▶) for Pr; Shen *et al.* (2011*c* ▶) for Dy; Shen *et al.* (2011*d* ▶) for Ce; Shen *et al.* (2011*e* ▶) for Ho; Shen *et al.* (2011*f* ▶) for Gd.
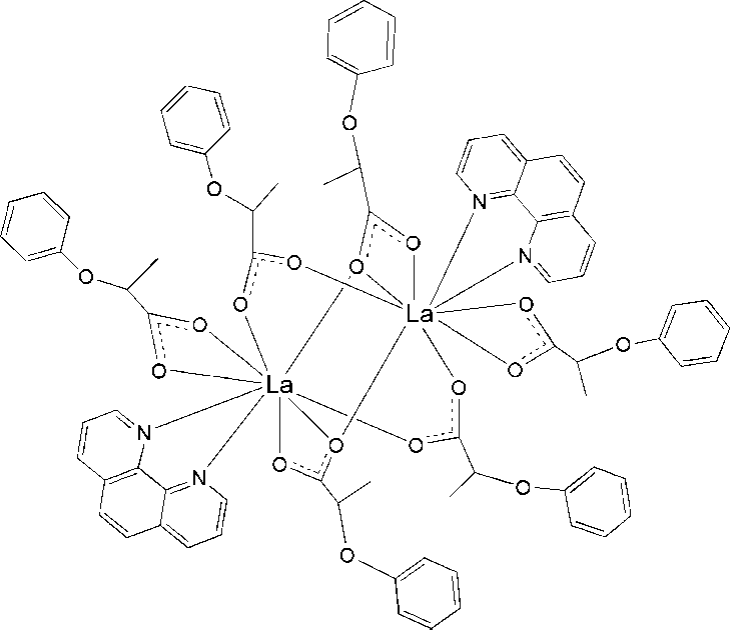


## Experimental

### 

#### Crystal data

[La_2_(C_9_H_9_O_3_)_6_(C_12_H_8_N_2_)_2_]
*M* *_r_* = 1629.20
Monoclinic, 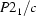

*a* = 11.5474 (2) Å
*b* = 25.9919 (4) Å
*c* = 13.9632 (2) Å
β = 119.9610 (1)°
*V* = 3630.85 (10) Å^3^
*Z* = 2
Mo *K*α radiation
μ = 1.23 mm^−1^
*T* = 296 K
0.24 × 0.16 × 0.08 mm

#### Data collection

Bruker APEXII CCD diffractometer
Absorption correction: multi-scan (*SADABS*; Sheldrick, 1996 ▶) *T*_min_ = 0.791, *T*_max_ = 0.907
49259 measured reflections
6395 independent reflections
5484 reflections with *I* > 2σ(*I*)
*R*_int_ = 0.032

#### Refinement

*R*[*F* ^2^ > 2σ(*F* ^2^)] = 0.022
*wR*(*F* ^2^) = 0.050
*S* = 1.05
6395 reflections
464 parameters
H-atom parameters constrained
Δρ_max_ = 0.42 e Å^−3^
Δρ_min_ = −0.27 e Å^−3^

### 

Data collection: *APEX2* (Bruker, 2006 ▶); cell refinement: *SAINT* (Bruker, 2006 ▶); data reduction: *SAINT*; program(s) used to solve structure: *SHELXS97* (Sheldrick, 2008 ▶); program(s) used to refine structure: *SHELXL97* (Sheldrick, 2008 ▶); molecular graphics: *XP* in *SHELXTL* (Sheldrick, 2008 ▶) and *DIAMOND* (Brandenburg, 2006 ▶); software used to prepare material for publication: *SHELXL97*.

## Supplementary Material

Crystal structure: contains datablock(s) I. DOI: 10.1107/S1600536811036117/wm2525sup1.cif

Structure factors: contains datablock(s) I. DOI: 10.1107/S1600536811036117/wm2525Isup2.hkl

Additional supplementary materials:  crystallographic information; 3D view; checkCIF report

## Figures and Tables

**Table 1:** Selected bond lengths (Å)

La1—O4^i^	2.4589 (14)
La1—O2	2.4770 (15)
La1—O1^i^	2.5054 (14)
La1—O7	2.5553 (16)
La1—O8	2.5722 (15)
La1—O5	2.5956 (14)
La1—N2	2.6642 (17)
La1—O4	2.7108 (14)
La1—N1	2.7179 (17)

Symmetry code: (i) 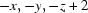.

**Fig. 1.**
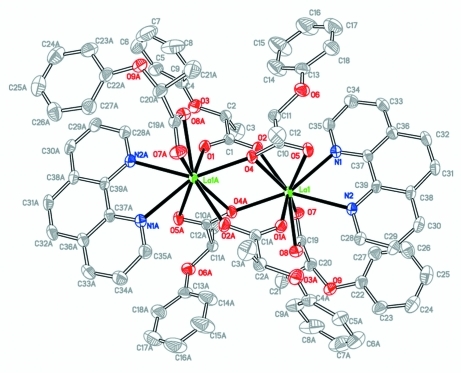
The molecular structure of the title complex, showing the atom-labelling scheme. Displacement ellipsoids are drawn at the 30% probability level.

**Fig. 2.**
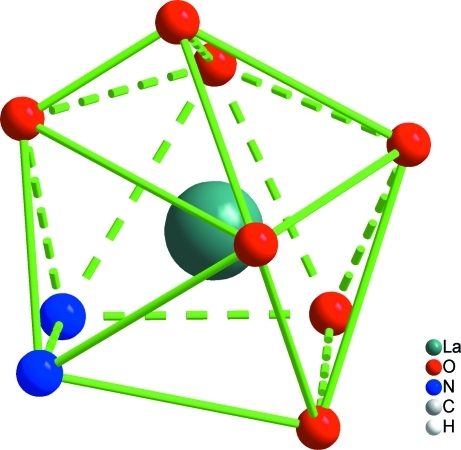
The coordination environment of the La(III) atom.

## supplementary crystallographic information

### Comment

The group of phenoxyalkanoic acids includes a considerable number of important herbicides. The desired biological activity is largely dependent on the length of the carbon chain of the alkanoic acid, the nature of the phenoxy group, and the position of its attachment to the carbon chain (Markus & Buser, 1997). The structures of 2-phenoxypropionic acid (H*L*) complexes coupled with their special functionality catched our interest. Here, we describe the La^III^ title complex, (I).

The structure of complex (I) is shown in Fig. 1 and the coordination environment of La(III) is shown in Fig. 2. The dimeric title compound (I) is centrosymmetric and is comprised of six *L* anions and two phenanthroline ligands. The two La(III) ions are linked together by four *L* groups through their bi- and tri-dentate bridging modes, forming a dimeric unit. The distance between two La(III) ions in the dimeric unit is 4.1302 (2) Å. Each La(III) ion is coordinated by nine atoms, five of which are oxygen atoms from the bridging carboxylates, two oxygen atoms from the bidentate chelating carboxylate group, and two nitrogen atoms from a 1,10-phenanthroline molecule. The Gd(III) ion adopts a distorted monocapped square antiprisatic geometry (Fig. 2). The La—O distances are within the range 2.4589 (18)–2.7108 (14) Å, and the La—N distances range from 2.6642 (17)–2.7179 (17) Å, all of which are within the range of those of other nine-coordinated La^III^ complexes with carboxylic donor ligands and 1,10-phenanthroline (Li *et al.*, 2010) (Table 1).

For isotypic structures, see: for Tb (Shen *et al.*, 2011*a*), for Pr (Shen *et al.*, 2011*b*), for Dy (Shen *et al.*, 2011*c*), for Ce (Shen *et al.*, 2011*d*), for Ho (Shen *et al.*, 2011*e*), for Gd (Shen *et al.*, 2011*f*).

### Experimental

Reagents and solvents used were of commercially available quality and without purified before use. 2-Phenoxypropionic acid (1.5 mmol), La(NO_3_)_3_^.^6H_2_O (0.5 mmol) and 1,10-phenanthroline (0.5 mmol) were dissolved in 20 ml enthanol, then 10 ml water were added to the above solution. The mixed solution was stirred for 12 h at room temperature. Finally, the deposit was filtered off and the colourless solution was kept in the open air. Colourless crystals were obtained after several days.

### Refinement

The structure was solved by direct methods and successive Fourier difference synthesis. The H atoms bonded to C and N atoms were positioned geometrically and refined using a riding model [aliphatic C—H =0.96 Å (*U*_iso_(H) = 1.5*U*_eq_(C)), aromatic C—H = 0.93 Å (*U*_iso_(H) = 1.2*U*_eq_(C))].

### Crystal data

**Table:**

[La_2_(C_9_H_9_O_3_)_6_(C_12_H_8_N_2_)_2_]	*F*(000) = 1648
*M**_r_* = 1629.20	*D*_x_ = 1.490 Mg m^−^^3^
Monoclinic, *P*2_1_/*c*	Mo *K*α radiation, λ = 0.71073 Å
Hall symbol: -P 2ybc	Cell parameters from 9919 reflections
*a* = 11.5474 (2) Å	θ = 1.6–25.0°
*b* = 25.9919 (4) Å	µ = 1.23 mm^−^^1^
*c* = 13.9632 (2) Å	*T* = 296 K
β = 119.9610 (1)°	Block, colourless
*V* = 3630.85 (10) Å^3^	0.24 × 0.16 × 0.08 mm
*Z* = 2	

### Data collection

**Table:**

Bruker APEXII CCD diffractometer	6395 independent reflections
Radiation source: fine-focus sealed tube	5484 reflections with *I* > 2σ(*I*)
graphite	*R*_int_ = 0.032
phi and ω scans	θ_max_ = 25.0°, θ_min_ = 1.6°
Absorption correction: multi-scan (*SADABS*; Sheldrick, 1996)	*h* = −13→13
*T*_min_ = 0.791, *T*_max_ = 0.907	*k* = −30→30
49259 measured reflections	*l* = −16→16

### Refinement

**Table:**

Refinement on *F*^2^	Primary atom site location: structure-invariant direct methods
Least-squares matrix: full	Secondary atom site location: difference Fourier map
*R*[*F*^2^ > 2σ(*F*^2^)] = 0.022	Hydrogen site location: inferred from neighbouring sites
*wR*(*F*^2^) = 0.050	H-atom parameters constrained
*S* = 1.05	*w* = 1/[σ^2^(*F*_o_^2^) + (0.0188*P*)^2^ + 1.8205*P*] where *P* = (*F*_o_^2^ + 2*F*_c_^2^)/3
6395 reflections	(Δ/σ)_max_ = 0.001
464 parameters	Δρ_max_ = 0.42 e Å^−^^3^
0 restraints	Δρ_min_ = −0.27 e Å^−^^3^

### Special details

**Table:**

Geometry. All e.s.d.'s (except the e.s.d. in the dihedral angle between two l.s. planes) are estimated using the full covariance matrix. The cell e.s.d.'s are taken into account individually in the estimation of e.s.d.'s in distances, angles and torsion angles; correlations between e.s.d.'s in cell parameters are only used when they are defined by crystal symmetry. An approximate (isotropic) treatment of cell e.s.d.'s is used for estimating e.s.d.'s involving l.s. planes.
Refinement. Refinement of *F*^2^ against ALL reflections. The weighted *R*-factor *wR* and goodness of fit *S* are based on *F*^2^, conventional *R*-factors *R* are based on *F*, with *F* set to zero for negative *F*^2^. The threshold expression of *F*^2^ > σ(*F*^2^) is used only for calculating *R*-factors(gt) *etc*. and is not relevant to the choice of reflections for refinement. *R*-factors based on *F*^2^ are statistically about twice as large as those based on *F*, and *R*- factors based on ALL data will be even larger.

### Fractional atomic coordinates and isotropic or equivalent isotropic displacement parameters (Å^2^)

**Table:**

	*x*	*y*	*z*	*U*_iso_*/*U*_eq_	
La1	−0.045539 (11)	0.002779 (4)	0.837031 (9)	0.03066 (5)	
O1	0.16892 (15)	−0.07028 (5)	1.12124 (12)	0.0432 (4)	
O2	0.08493 (16)	−0.07409 (6)	0.93843 (13)	0.0477 (4)	
O3	0.32482 (17)	−0.15522 (6)	1.15316 (15)	0.0585 (5)	
O4	−0.12480 (14)	−0.03224 (6)	0.97813 (12)	0.0402 (3)	
O5	−0.26299 (14)	−0.04358 (6)	0.80046 (12)	0.0419 (4)	
O6	−0.41270 (15)	−0.11094 (6)	0.84679 (14)	0.0508 (4)	
O7	0.13101 (18)	0.01594 (6)	0.77999 (15)	0.0539 (4)	
O8	0.03697 (16)	0.08693 (6)	0.79428 (13)	0.0469 (4)	
O9	0.10207 (19)	0.13569 (6)	0.65043 (15)	0.0611 (5)	
N1	−0.13093 (18)	−0.06866 (7)	0.67346 (15)	0.0384 (4)	
N2	−0.22177 (17)	0.03015 (7)	0.63058 (14)	0.0386 (4)	
C1	0.1576 (2)	−0.08966 (8)	1.03531 (18)	0.0388 (5)	
C2	0.2392 (2)	−0.13775 (9)	1.0442 (2)	0.0469 (6)	
H2A	0.1767	−0.1655	1.0027	0.056*	
C3	0.3249 (3)	−0.12750 (12)	0.9929 (3)	0.0753 (9)	
H3A	0.3746	−0.1579	0.9976	0.113*	
H3B	0.3857	−0.0999	1.0316	0.113*	
H3C	0.2690	−0.1182	0.9167	0.113*	
C4	0.2729 (3)	−0.18288 (9)	1.2063 (2)	0.0562 (7)	
C5	0.3679 (4)	−0.20955 (11)	1.2977 (3)	0.0836 (10)	
H5A	0.4571	−0.2090	1.3158	0.100*	
C6	0.3297 (6)	−0.23655 (16)	1.3608 (4)	0.1232 (17)	
H6A	0.3934	−0.2543	1.4226	0.148*	
C7	0.1985 (7)	−0.23783 (16)	1.3342 (5)	0.129 (2)	
H7A	0.1733	−0.2566	1.3775	0.155*	
C8	0.1053 (5)	−0.21172 (13)	1.2447 (4)	0.1064 (14)	
H8A	0.0164	−0.2124	1.2275	0.128*	
C9	0.1410 (3)	−0.18421 (10)	1.1789 (3)	0.0700 (8)	
H9A	0.0766	−0.1668	1.1169	0.084*	
C10	−0.2338 (2)	−0.04873 (7)	0.89805 (17)	0.0336 (5)	
C11	−0.3315 (2)	−0.07362 (8)	0.92705 (19)	0.0426 (5)	
H11A	−0.2818	−0.0901	0.9998	0.051*	
C12	−0.4243 (3)	−0.03338 (11)	0.9295 (2)	0.0622 (7)	
H12A	−0.4838	−0.0492	0.9502	0.093*	
H12B	−0.4753	−0.0182	0.8576	0.093*	
H12C	−0.3728	−0.0072	0.9824	0.093*	
C13	−0.3508 (3)	−0.15388 (9)	0.8368 (2)	0.0543 (6)	
C14	−0.2204 (3)	−0.16687 (11)	0.9085 (3)	0.0957 (12)	
H14A	−0.1666	−0.1456	0.9680	0.115*	
C15	−0.1698 (4)	−0.21173 (13)	0.8913 (4)	0.1262 (18)	
H15A	−0.0817	−0.2207	0.9406	0.151*	
C16	−0.2450 (4)	−0.24310 (13)	0.8045 (4)	0.1125 (14)	
H16A	−0.2092	−0.2733	0.7945	0.135*	
C17	−0.3740 (4)	−0.22960 (13)	0.7319 (3)	0.0987 (12)	
H17A	−0.4260	−0.2504	0.6711	0.118*	
C18	−0.4282 (3)	−0.18511 (11)	0.7482 (3)	0.0731 (8)	
H18A	−0.5167	−0.1765	0.6992	0.088*	
C19	0.1091 (2)	0.06325 (9)	0.76581 (18)	0.0423 (5)	
C20	0.1801 (3)	0.09372 (10)	0.7160 (2)	0.0541 (6)	
H20A	0.2012	0.0708	0.6711	0.065*	
C21	0.3084 (3)	0.11672 (13)	0.8086 (3)	0.0788 (9)	
H21A	0.3536	0.1354	0.7776	0.118*	
H21B	0.3653	0.0896	0.8551	0.118*	
H21C	0.2874	0.1396	0.8517	0.118*	
C22	−0.0049 (3)	0.12582 (10)	0.5477 (2)	0.0522 (6)	
C23	−0.0762 (3)	0.16847 (11)	0.4894 (3)	0.0671 (8)	
H23A	−0.0526	0.2008	0.5220	0.081*	
C24	−0.1811 (3)	0.16356 (14)	0.3843 (3)	0.0787 (9)	
H24A	−0.2277	0.1927	0.3456	0.094*	
C25	−0.2186 (3)	0.11626 (15)	0.3350 (3)	0.0769 (9)	
H25A	−0.2904	0.1131	0.2635	0.092*	
C26	−0.1482 (3)	0.07340 (13)	0.3930 (3)	0.0713 (8)	
H26A	−0.1726	0.0411	0.3601	0.086*	
C27	−0.0417 (3)	0.07780 (11)	0.4999 (2)	0.0587 (7)	
H27A	0.0045	0.0487	0.5390	0.070*	
C28	−0.2633 (2)	0.07851 (9)	0.60777 (19)	0.0467 (6)	
H28A	−0.2187	0.1033	0.6621	0.056*	
C29	−0.3703 (2)	0.09422 (10)	0.5067 (2)	0.0506 (6)	
H29A	−0.3956	0.1286	0.4944	0.061*	
C30	−0.4368 (2)	0.05872 (10)	0.4267 (2)	0.0507 (6)	
H30A	−0.5090	0.0685	0.3591	0.061*	
C31	−0.4619 (2)	−0.03281 (10)	0.36706 (19)	0.0511 (6)	
H31A	−0.5358	−0.0248	0.2990	0.061*	
C32	−0.4192 (2)	−0.08167 (11)	0.3885 (2)	0.0536 (6)	
H32A	−0.4646	−0.1069	0.3354	0.064*	
C33	−0.2550 (3)	−0.14596 (10)	0.5166 (2)	0.0586 (7)	
H33A	−0.2959	−0.1721	0.4649	0.070*	
C34	−0.1459 (3)	−0.15638 (10)	0.6167 (2)	0.0604 (7)	
H34A	−0.1111	−0.1895	0.6339	0.072*	
C35	−0.0873 (2)	−0.11664 (9)	0.6929 (2)	0.0501 (6)	
H35A	−0.0133	−0.1243	0.7612	0.060*	
C36	−0.3052 (2)	−0.09571 (9)	0.49169 (19)	0.0451 (5)	
C37	−0.2389 (2)	−0.05781 (8)	0.57307 (17)	0.0373 (5)	
C38	−0.3965 (2)	0.00714 (9)	0.44617 (18)	0.0419 (5)	
C39	−0.2861 (2)	−0.00566 (8)	0.55023 (17)	0.0363 (5)	

### Atomic displacement parameters (Å^2^)

**Table:**

	*U*^11^	*U*^22^	*U*^33^	*U*^12^	*U*^13^	*U*^23^
La1	0.02874 (7)	0.03534 (7)	0.02364 (7)	−0.00036 (5)	0.00988 (5)	0.00054 (5)
O1	0.0491 (9)	0.0409 (8)	0.0360 (9)	0.0096 (7)	0.0185 (8)	0.0034 (7)
O2	0.0526 (10)	0.0480 (9)	0.0327 (9)	0.0138 (7)	0.0139 (8)	0.0046 (7)
O3	0.0460 (10)	0.0539 (10)	0.0587 (11)	0.0106 (8)	0.0135 (9)	0.0102 (9)
O4	0.0347 (8)	0.0473 (9)	0.0290 (8)	−0.0083 (7)	0.0087 (7)	−0.0017 (6)
O5	0.0340 (8)	0.0573 (9)	0.0292 (8)	−0.0077 (7)	0.0117 (7)	−0.0026 (7)
O6	0.0362 (9)	0.0469 (9)	0.0593 (11)	−0.0074 (7)	0.0163 (8)	−0.0006 (8)
O7	0.0622 (11)	0.0459 (9)	0.0688 (12)	0.0035 (8)	0.0441 (10)	0.0082 (8)
O8	0.0475 (10)	0.0422 (8)	0.0525 (10)	−0.0009 (7)	0.0262 (9)	0.0037 (7)
O9	0.0708 (12)	0.0526 (10)	0.0493 (11)	−0.0125 (9)	0.0222 (10)	0.0109 (8)
N1	0.0363 (10)	0.0429 (10)	0.0331 (10)	0.0011 (8)	0.0152 (9)	−0.0019 (8)
N2	0.0360 (10)	0.0435 (10)	0.0295 (10)	0.0013 (8)	0.0110 (8)	0.0021 (8)
C1	0.0376 (12)	0.0376 (11)	0.0374 (13)	0.0020 (9)	0.0159 (11)	0.0005 (10)
C2	0.0435 (14)	0.0440 (13)	0.0443 (14)	0.0097 (10)	0.0153 (11)	0.0007 (10)
C3	0.067 (2)	0.088 (2)	0.081 (2)	0.0260 (16)	0.0446 (18)	0.0084 (17)
C4	0.0717 (19)	0.0327 (12)	0.0570 (17)	0.0077 (12)	0.0268 (15)	0.0021 (11)
C5	0.100 (3)	0.0502 (17)	0.069 (2)	0.0046 (16)	0.0186 (19)	0.0100 (15)
C6	0.186 (5)	0.074 (3)	0.078 (3)	0.009 (3)	0.042 (4)	0.024 (2)
C7	0.240 (7)	0.066 (2)	0.133 (4)	0.007 (4)	0.131 (5)	0.022 (3)
C8	0.151 (4)	0.056 (2)	0.162 (4)	0.000 (2)	0.116 (4)	0.008 (2)
C9	0.080 (2)	0.0436 (15)	0.090 (2)	0.0062 (14)	0.0455 (19)	0.0099 (14)
C10	0.0314 (11)	0.0325 (10)	0.0334 (12)	0.0006 (8)	0.0135 (10)	−0.0011 (9)
C11	0.0389 (13)	0.0484 (13)	0.0390 (13)	−0.0062 (10)	0.0184 (11)	0.0006 (10)
C12	0.0552 (17)	0.0738 (18)	0.0723 (19)	0.0006 (14)	0.0430 (16)	−0.0040 (15)
C13	0.0465 (15)	0.0394 (13)	0.0679 (18)	−0.0075 (11)	0.0218 (14)	0.0048 (12)
C14	0.063 (2)	0.0526 (17)	0.116 (3)	0.0084 (15)	0.004 (2)	−0.0131 (18)
C15	0.080 (3)	0.062 (2)	0.174 (4)	0.0191 (19)	0.017 (3)	−0.026 (3)
C16	0.105 (3)	0.056 (2)	0.152 (4)	0.013 (2)	0.046 (3)	−0.016 (2)
C17	0.110 (3)	0.064 (2)	0.103 (3)	−0.009 (2)	0.039 (3)	−0.024 (2)
C18	0.0654 (19)	0.0618 (18)	0.074 (2)	−0.0080 (15)	0.0214 (17)	−0.0058 (15)
C19	0.0378 (13)	0.0485 (14)	0.0357 (13)	−0.0046 (10)	0.0147 (11)	0.0054 (10)
C20	0.0533 (16)	0.0600 (15)	0.0503 (15)	−0.0080 (12)	0.0268 (13)	0.0108 (12)
C21	0.0563 (18)	0.106 (2)	0.066 (2)	−0.0285 (17)	0.0246 (16)	0.0152 (18)
C22	0.0576 (16)	0.0595 (15)	0.0455 (15)	−0.0068 (12)	0.0303 (13)	0.0088 (12)
C23	0.0659 (19)	0.0594 (17)	0.074 (2)	−0.0042 (14)	0.0337 (17)	0.0160 (15)
C24	0.064 (2)	0.092 (2)	0.074 (2)	0.0104 (18)	0.0304 (18)	0.0323 (19)
C25	0.0604 (19)	0.117 (3)	0.0519 (18)	0.0037 (19)	0.0267 (16)	0.0117 (19)
C26	0.071 (2)	0.091 (2)	0.0589 (19)	−0.0108 (17)	0.0373 (17)	−0.0167 (17)
C27	0.0628 (17)	0.0614 (17)	0.0549 (17)	0.0012 (13)	0.0317 (15)	0.0010 (13)
C28	0.0485 (14)	0.0478 (13)	0.0374 (13)	0.0024 (11)	0.0166 (12)	0.0028 (10)
C29	0.0478 (14)	0.0536 (14)	0.0445 (15)	0.0115 (12)	0.0187 (12)	0.0121 (12)
C30	0.0407 (14)	0.0697 (16)	0.0342 (13)	0.0069 (12)	0.0130 (11)	0.0142 (12)
C31	0.0400 (14)	0.0760 (18)	0.0274 (12)	−0.0080 (12)	0.0093 (11)	−0.0001 (12)
C32	0.0475 (15)	0.0706 (17)	0.0354 (14)	−0.0171 (13)	0.0152 (12)	−0.0148 (12)
C33	0.0629 (17)	0.0556 (15)	0.0531 (17)	−0.0137 (13)	0.0257 (15)	−0.0199 (13)
C34	0.0699 (19)	0.0458 (14)	0.0605 (18)	0.0034 (13)	0.0289 (16)	−0.0076 (12)
C35	0.0500 (15)	0.0506 (14)	0.0429 (14)	0.0051 (11)	0.0181 (12)	−0.0009 (11)
C36	0.0434 (13)	0.0544 (14)	0.0378 (13)	−0.0100 (11)	0.0205 (11)	−0.0084 (11)
C37	0.0328 (12)	0.0500 (12)	0.0311 (12)	−0.0052 (9)	0.0174 (10)	−0.0032 (9)
C38	0.0344 (12)	0.0603 (14)	0.0289 (11)	−0.0032 (10)	0.0144 (10)	0.0033 (10)
C39	0.0319 (11)	0.0501 (12)	0.0269 (11)	−0.0026 (9)	0.0146 (9)	0.0026 (9)

### Geometric parameters (Å, °)

**Table:**

La1—O4^i^	2.4589 (14)	C12—H12B	0.9600
La1—O2	2.4770 (15)	C12—H12C	0.9600
La1—O1^i^	2.5054 (14)	C13—C18	1.372 (4)
La1—O7	2.5553 (16)	C13—C14	1.371 (4)
La1—O8	2.5722 (15)	C14—C15	1.378 (4)
La1—O5	2.5956 (14)	C14—H14A	0.9300
La1—N2	2.6642 (17)	C15—C16	1.355 (5)
La1—O4	2.7108 (14)	C15—H15A	0.9300
La1—N1	2.7179 (17)	C16—C17	1.365 (5)
La1—C19	2.902 (2)	C16—H16A	0.9300
La1—C10	3.016 (2)	C17—C18	1.387 (4)
La1—La1^i^	4.1302 (2)	C17—H17A	0.9300
O1—C1	1.247 (3)	C18—H18A	0.9300
O1—La1^i^	2.5054 (14)	C19—C20	1.534 (3)
O2—C1	1.251 (3)	C20—C21	1.520 (4)
O3—C4	1.369 (3)	C20—H20A	0.9800
O3—C2	1.413 (3)	C21—H21A	0.9600
O4—C10	1.270 (2)	C21—H21B	0.9600
O4—La1^i^	2.4589 (14)	C21—H21C	0.9600
O5—C10	1.237 (2)	C22—C23	1.378 (4)
O6—C13	1.369 (3)	C22—C27	1.378 (4)
O6—C11	1.423 (3)	C23—C24	1.365 (4)
O7—C19	1.251 (3)	C23—H23A	0.9300
O8—C19	1.250 (3)	C24—C25	1.369 (5)
O9—C22	1.371 (3)	C24—H24A	0.9300
O9—C20	1.420 (3)	C25—C26	1.379 (4)
N1—C35	1.322 (3)	C25—H25A	0.9300
N1—C37	1.362 (3)	C26—C27	1.386 (4)
N2—C28	1.326 (3)	C26—H26A	0.9300
N2—C39	1.358 (3)	C27—H27A	0.9300
C1—C2	1.532 (3)	C28—C29	1.394 (3)
C2—C3	1.508 (4)	C28—H28A	0.9300
C2—H2A	0.9800	C29—C30	1.354 (3)
C3—H3A	0.9600	C29—H29A	0.9300
C3—H3B	0.9600	C30—C38	1.401 (3)
C3—H3C	0.9600	C30—H30A	0.9300
C4—C9	1.373 (4)	C31—C32	1.341 (4)
C4—C5	1.383 (4)	C31—C38	1.427 (3)
C5—C6	1.360 (6)	C31—H31A	0.9300
C5—H5A	0.9300	C32—C36	1.431 (3)
C6—C7	1.369 (7)	C32—H32A	0.9300
C6—H6A	0.9300	C33—C34	1.362 (4)
C7—C8	1.355 (6)	C33—C36	1.401 (4)
C7—H7A	0.9300	C33—H33A	0.9300
C8—C9	1.379 (4)	C34—C35	1.392 (3)
C8—H8A	0.9300	C34—H34A	0.9300
C9—H9A	0.9300	C35—H35A	0.9300
C10—C11	1.520 (3)	C36—C37	1.407 (3)
C11—C12	1.510 (3)	C37—C39	1.436 (3)
C11—H11A	0.9800	C38—C39	1.413 (3)
C12—H12A	0.9600		
			
O4^i^—La1—O2	73.25 (5)	C9—C8—H8A	119.7
O4^i^—La1—O1^i^	77.73 (5)	C4—C9—C8	119.1 (3)
O2—La1—O1^i^	133.32 (5)	C4—C9—H9A	120.5
O4^i^—La1—O7	86.62 (5)	C8—C9—H9A	120.5
O2—La1—O7	86.21 (5)	O5—C10—O4	122.56 (19)
O1^i^—La1—O7	127.85 (5)	O5—C10—C11	120.63 (19)
O4^i^—La1—O8	77.52 (5)	O4—C10—C11	116.77 (19)
O2—La1—O8	128.94 (5)	O5—C10—La1	58.59 (10)
O1^i^—La1—O8	77.18 (5)	O4—C10—La1	63.98 (10)
O7—La1—O8	50.81 (5)	C11—C10—La1	178.48 (15)
O4^i^—La1—O5	122.63 (5)	O6—C11—C12	107.11 (19)
O2—La1—O5	89.45 (5)	O6—C11—C10	111.40 (18)
O1^i^—La1—O5	76.37 (5)	C12—C11—C10	109.92 (19)
O7—La1—O5	147.66 (5)	O6—C11—H11A	109.5
O8—La1—O5	141.55 (5)	C12—C11—H11A	109.5
O4^i^—La1—N2	146.20 (5)	C10—C11—H11A	109.5
O2—La1—N2	138.74 (5)	C11—C12—H12A	109.5
O1^i^—La1—N2	81.31 (5)	C11—C12—H12B	109.5
O7—La1—N2	85.65 (6)	H12A—C12—H12B	109.5
O8—La1—N2	72.13 (5)	C11—C12—H12C	109.5
O5—La1—N2	76.70 (5)	H12A—C12—H12C	109.5
O4^i^—La1—O4	74.05 (5)	H12B—C12—H12C	109.5
O2—La1—O4	69.11 (5)	O6—C13—C18	115.9 (2)
O1^i^—La1—O4	68.19 (5)	O6—C13—C14	124.5 (3)
O7—La1—O4	152.00 (5)	C18—C13—C14	119.7 (3)
O8—La1—O4	138.83 (5)	C13—C14—C15	119.2 (3)
O5—La1—O4	48.89 (4)	C13—C14—H14A	120.4
N2—La1—O4	121.56 (5)	C15—C14—H14A	120.4
O4^i^—La1—N1	149.22 (5)	C16—C15—C14	121.8 (4)
O2—La1—N1	77.94 (5)	C16—C15—H15A	119.1
O1^i^—La1—N1	131.61 (5)	C14—C15—H15A	119.1
O7—La1—N1	80.83 (5)	C15—C16—C17	118.9 (3)
O8—La1—N1	114.01 (5)	C15—C16—H16A	120.5
O5—La1—N1	66.93 (5)	C17—C16—H16A	120.5
N2—La1—N1	60.83 (5)	C16—C17—C18	120.5 (3)
O4—La1—N1	105.60 (5)	C16—C17—H17A	119.8
O4^i^—La1—C19	83.36 (6)	C18—C17—H17A	119.8
O2—La1—C19	109.27 (6)	C13—C18—C17	119.8 (3)
O1^i^—La1—C19	102.66 (6)	C13—C18—H18A	120.1
O7—La1—C19	25.50 (6)	C17—C18—H18A	120.1
O8—La1—C19	25.49 (6)	O8—C19—O7	123.2 (2)
O5—La1—C19	152.18 (6)	O8—C19—C20	118.8 (2)
N2—La1—C19	75.69 (6)	O7—C19—C20	117.9 (2)
O4—La1—C19	156.89 (6)	O8—C19—La1	62.32 (11)
N1—La1—C19	96.27 (6)	O7—C19—La1	61.55 (12)
O4^i^—La1—C10	98.82 (5)	C20—C19—La1	174.12 (16)
O2—La1—C10	78.40 (5)	O9—C20—C21	106.3 (2)
O1^i^—La1—C10	70.81 (5)	O9—C20—C19	112.2 (2)
O7—La1—C10	161.34 (5)	C21—C20—C19	109.4 (2)
O8—La1—C10	147.77 (5)	O9—C20—H20A	109.6
O5—La1—C10	24.00 (5)	C21—C20—H20A	109.6
N2—La1—C10	98.94 (5)	C19—C20—H20A	109.6
O4—La1—C10	24.90 (5)	C20—C21—H21A	109.5
N1—La1—C10	85.64 (5)	C20—C21—H21B	109.5
C19—La1—C10	172.32 (6)	H21A—C21—H21B	109.5
O4^i^—La1—La1^i^	39.13 (3)	C20—C21—H21C	109.5
O2—La1—La1^i^	66.08 (4)	H21A—C21—H21C	109.5
O1^i^—La1—La1^i^	68.26 (3)	H21B—C21—H21C	109.5
O7—La1—La1^i^	122.98 (4)	O9—C22—C23	115.3 (2)
O8—La1—La1^i^	111.44 (4)	O9—C22—C27	125.1 (2)
O5—La1—La1^i^	83.66 (3)	C23—C22—C27	119.6 (3)
N2—La1—La1^i^	146.95 (4)	C24—C23—C22	120.5 (3)
O4—La1—La1^i^	34.92 (3)	C24—C23—H23A	119.8
N1—La1—La1^i^	133.47 (4)	C22—C23—H23A	119.8
C19—La1—La1^i^	122.33 (5)	C23—C24—C25	120.8 (3)
C10—La1—La1^i^	59.73 (4)	C23—C24—H24A	119.6
C1—O1—La1^i^	135.11 (14)	C25—C24—H24A	119.6
C1—O2—La1	140.12 (14)	C24—C25—C26	119.0 (3)
C4—O3—C2	119.5 (2)	C24—C25—H25A	120.5
C10—O4—La1^i^	161.96 (13)	C26—C25—H25A	120.5
C10—O4—La1	91.12 (12)	C25—C26—C27	120.8 (3)
La1^i^—O4—La1	105.95 (5)	C25—C26—H26A	119.6
C10—O5—La1	97.42 (12)	C27—C26—H26A	119.6
C13—O6—C11	117.61 (18)	C22—C27—C26	119.3 (3)
C19—O7—La1	92.95 (14)	C22—C27—H27A	120.4
C19—O8—La1	92.19 (13)	C26—C27—H27A	120.4
C22—O9—C20	118.8 (2)	N2—C28—C29	123.5 (2)
C35—N1—C37	117.79 (19)	N2—C28—H28A	118.2
C35—N1—La1	122.08 (15)	C29—C28—H28A	118.2
C37—N1—La1	119.01 (13)	C30—C29—C28	119.1 (2)
C28—N2—C39	117.91 (19)	C30—C29—H29A	120.4
C28—N2—La1	120.14 (15)	C28—C29—H29A	120.4
C39—N2—La1	121.20 (13)	C29—C30—C38	119.7 (2)
O1—C1—O2	127.0 (2)	C29—C30—H30A	120.1
O1—C1—C2	119.1 (2)	C38—C30—H30A	120.1
O2—C1—C2	113.88 (19)	C32—C31—C38	121.5 (2)
O3—C2—C3	107.2 (2)	C32—C31—H31A	119.2
O3—C2—C1	114.88 (19)	C38—C31—H31A	119.2
C3—C2—C1	109.9 (2)	C31—C32—C36	121.1 (2)
O3—C2—H2A	108.2	C31—C32—H32A	119.4
C3—C2—H2A	108.2	C36—C32—H32A	119.4
C1—C2—H2A	108.2	C34—C33—C36	119.7 (2)
C2—C3—H3A	109.5	C34—C33—H33A	120.1
C2—C3—H3B	109.5	C36—C33—H33A	120.1
H3A—C3—H3B	109.5	C33—C34—C35	119.0 (2)
C2—C3—H3C	109.5	C33—C34—H34A	120.5
H3A—C3—H3C	109.5	C35—C34—H34A	120.5
H3B—C3—H3C	109.5	N1—C35—C34	123.6 (2)
O3—C4—C9	126.0 (2)	N1—C35—H35A	118.2
O3—C4—C5	113.8 (3)	C34—C35—H35A	118.2
C9—C4—C5	120.2 (3)	C33—C36—C37	117.5 (2)
C6—C5—C4	119.4 (4)	C33—C36—C32	123.1 (2)
C6—C5—H5A	120.3	C37—C36—C32	119.4 (2)
C4—C5—H5A	120.3	N1—C37—C36	122.4 (2)
C5—C6—C7	120.6 (4)	N1—C37—C39	118.35 (19)
C5—C6—H6A	119.7	C36—C37—C39	119.3 (2)
C7—C6—H6A	119.7	C30—C38—C39	117.8 (2)
C8—C7—C6	120.0 (4)	C30—C38—C31	123.4 (2)
C8—C7—H7A	120.0	C39—C38—C31	118.8 (2)
C6—C7—H7A	120.0	N2—C39—C38	121.9 (2)
C7—C8—C9	120.6 (4)	N2—C39—C37	118.24 (19)
C7—C8—H8A	119.7	C38—C39—C37	119.85 (19)

Symmetry codes: (i) −*x*, −*y*, −*z*+2.
